# Prevalence of Childhood Trauma Among Women with Schizophrenia: A Cross-Sectional Study

**DOI:** 10.1192/j.eurpsy.2024.1548

**Published:** 2024-08-27

**Authors:** S. Walha, I. Chaari, R. Ben Jmaa, A. Ksentini, L. Aribi, F. Charfeddine, N. Mseddi, J. Aloulou

**Affiliations:** Psychiatry « B » department, Hedi Chaker University Hospital, Sfax, Tunisia

## Abstract

**Introduction:**

Schizophrenia is a complex and multifactorial disorder believed to arise from the interplay between genetic factors and environmental influences. Among these environmental factors, childhood trauma stands out as a significant contributor to the onset of schizophrenia in adulthood.

**Objectives:**

The objective of this study was to assess the occurrence rates of physical, emotional, and sexual abuse, as well as physical and emotional neglect in a group of Tunisian women diagnosed with schizophrenia spectrum disorders.

**Methods:**

We conducted a descriptive cross-sectional study among stabilized female patients with schizophrenia or schizoaffective disorder, in the ‘B’ psychiatry department at Hedi Chaker University Hospital in Sfax, Tunisia, from May to June 2023. We administered the 28-item Childhood Trauma Questionnaire (CTQ).

**Results:**

In this study, 41 female patients were enrolled, with 65.9% diagnosed with schizophrenia and 34.2% with schizoaffective disorder. The average age of participants was 49.19 years, ranging from 17 to 79. The mean score on the Childhood Trauma Questionnaire (CTQ) was 56.34, with scores ranging from 43 to 98. Emotional neglect was the most prevalent form of trauma, reported by 40 patients (97.6%). Following emotional neglect, physical abuse was found in 16 patients (39%), sexual abuse in 10 patients (24.4%), emotional abuse in 6 patients (14.6%), and physical neglect in 2 patients (4.9%).

**Conclusions:**

Based on these findings, our recommendation is to establish government-operated facilities that offer emotional and psychological support, legal assistance, parenting guidance, and medical monitoring in collaboration with educational institutions and social and child protection services for those in need.

**Disclosure of Interest:**

None Declared

